# Global strategies for the diffusion of robotic surgery

**DOI:** 10.1590/0102-67202025000039e1908

**Published:** 2025-11-10

**Authors:** Francisco TUSTUMI, Louisa BOLM, Rodrigo Camargo Leão EDELMUTH, Felipe Antonio Boff MAEGAWA, Wellington ANDRAUS, Paulo HERMAN, Tyler MCKECHNIE, Allan TSUNG, Sarah SAMREEN, Ryan MERKOW, Nigel D’SOUZA, Syed Nabeel ZAFAR, Giovanna Mennitti SHIMODA, Nelson WOLOSKER, Yoshikuni KAWAGUCHI, Georgios TSOULFAS, Eduardo Esteban MONTALVO-JAVE, Vikas DUDEJA, Puja Gaur KHAITAN, Sajid KHAN

**Affiliations:** 1 Hospital Israelita Albert Einstein, Department of Health Sciences – São Paulo (SP), Brazil.; 2 Universidade de São Paulo, Department of Gastroenterology – São Paulo (SP), Brazil.; 3 University Medical Center Schleswig-Holstein, Department of Surgery, Campus Luebeck – Luebeck, Germany.; 4 Emory University, Department of Surgery – Atlanta (GA), United States of America.; 5 McMaster University, Department of Surgery – Hamilton (ON), Canada.; 6 University of Virginia Health, Department of Surgery – Charlottesville (VA), United States of America.; 7 University of Texas Medical Branch, Department of Surgery – Galveston (TX), United States of America.; 8 University of Chicago, Surgical Implementation and Health Services Research Center, Department of Surgery – Chicago (IL), United States of America.; 9 University Hospital Southampton, Department of Coloproctology – Southampton, United Kingdom.; 10 University of Wisconsin-Madison, Department of Surgery – Madison (WI), United States of America.; 11 University of Tokyo, Department of Surgery – Tokyo, Japan.; 12 Aristotle University School of Medicine, Department of Transplantation Surgery – Thessaloniki, Greece.; 13 National Autonomous University of Mexico, Faculty of Medicine, Department of Surgery – Mexico City, Mexico.; 14 "Hospital General de Mexico," Dr. Eduardo Liceaga, Department of General Surgery, Hepato-Pancreato-Biliary Surgery – Mexico City, Mexico.; 15 Medica Sur Clinic & Foundation – Mexico City, Mexico.; 16 University of Iowa Health Care, Department of Surgery – Iowa City (IA), United States of America.; 17 Khalifa University, Sheikh Shakhbout Medical City, Gulf Medical University, Division of Thoracic Surgery, Department of Surgery – Abu Dhabi, United Arab Emirates.; 18 Yale School of Medicine, Hepato-Pancreato-Biliary and Mixed Tumors – New Haven (CT), United States of America.

**Keywords:** Robotic Surgical Procedures, Technology, Laparoscopy, Social Determinants of Health, Teaching, Procedimentos Cirúrgicos Robóticos, Tecnologia, Laparoscopia, Determinantes Sociais da Saúde, Ensino

## Abstract

**Background:**

The global adoption of robotic surgery has advanced rapidly in high-income countries, yet its diffusion remains limited in resource-constrained settings due to financial, infrastructural, and educational barriers. As surgical technology evolves, there is an urgent need to promote countries’ equitable access to robotic platforms worldwide.

**Aims:**

The aim of this study was to analyze global strategies employed to promote the diffusion of robotic surgery, with a particular focus on overcoming barriers in resource-limited settings, and to provide practical insights that can guide its equitable and sustainable implementation.

**Methods:**

This study is a multinational, policy-oriented integrative review conducted under the guidance of the Research Committee of the Society for Surgery of the Alimentary Tract in the USA (SSAT). The study integrates a bibliometric analysis, a literature review, and expert insights from diverse healthcare environments. Contributions were gathered from SSAT members.

**Results:**

Robotic platforms are predominantly concentrated in North America, Western Europe, and Eastern Asia, with the USA hosting nearly 60% of all installations. Research output is similarly skewed, with few countries and institutions producing most clinical trials. Key barriers to diffusion include high costs, lack of infrastructure, limited training capacity, regulatory hurdles, and resistance among surgeons. Facilitators include public–private partnerships, philanthropic support, technology transfer, simulation platforms, and curriculum integration by professional societies.

**Conclusions:**

Achieving global equity in robotic surgery requires coordinated action across research, education, clinical practice, policy, and infrastructure. Global cooperation and innovation in implementation strategies can help bridge the current disparities and promote safe, cost-effective surgical care in underserved regions, improving patient outcomes.

## ARTICLE HIGHLIGHTS

Robotic surgery adoption is concentrated in high-income locations, with major disparities in access worldwide.

Financial, infrastructural, and educational barriers limit diffusion in resource-constrained settings.

Coordinated global strategies are crucial for expanding safe and cost-effective robotic surgery to underserved regions.

## Summary

**CENTRAL MESSAGE:**

Robotic surgery is spreading quickly in wealthy countries but remains out of reach for many parts of the world. Our study reviewed global strategies for expanding access, with a focus on solutions for low-resource settings. We highlight the main barriers—costs, training, and infrastructure—and the strategies that can help, such as partnerships, education, and innovation.

**PERSPECTIVES:**

This study demonstrates how countries and institutions can collaborate to enhance the safety, affordability, and accessibility of robotic surgery. By sharing knowledge, supporting training, and creating fair policies, health systems can bring advanced surgical care to more patients worldwide.

## VISUAL ABSTRACT

**Figure:**
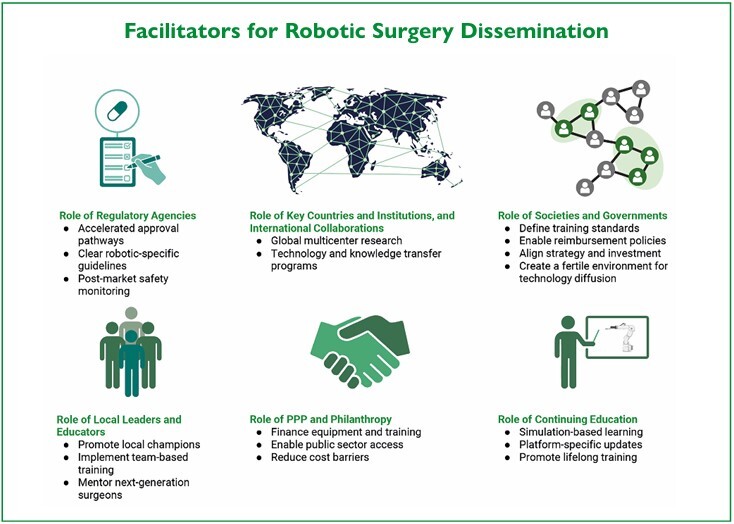

## RESUMO

**Racional:**

A adoção global da cirurgia robótica avançou rapidamente em países de alta renda, mas sua difusão permanece limitada em ambientes com menos recursos devido a barreiras financeiras, de infraestrutura e educacionais. À medida que a tecnologia cirúrgica evolui, há uma necessidade urgente de promover o acesso equitativo dos países às plataformas robóticas em todo o mundo.

**Objetivos:**

Analisar as estratégias globais empregadas para promover a difusão da cirurgia robótica, com foco particular na superação de barreiras em ambientes com recursos limitados, e fornecer *insights* práticos que possam orientar a sua implementação equitativa e sustentável.

**Métodos:**

Este estudo é uma revisão integrativa multinacional orientada a políticas, conduzida sob a orientação do Comitê de Pesquisa da Sociedade de Cirurgia do Trato Alimentar nos Estados Unidos da América (SSAT, EUA). O estudo integra uma análise bibliométrica, uma revisão de literatura e insights de especialistas de diversos ambientes de saúde. As contribuições foram coletadas dos membros da SSAT.

**Resultados:**

As plataformas robóticas concentram-se predominantemente na América do Norte, Europa Ocidental e Ásia Oriental, com os EUA abrigando quase 60% de todas as instalações. A produção científica é igualmente distorcida, com poucos países e instituições produzindo a maioria dos ensaios clínicos. As principais barreiras à difusão incluem altos custos, falta de infraestrutura, capacidade limitada de treinamento, obstáculos regulatórios e resistência entre cirurgiões. Os facilitadores incluem parcerias público-privadas, apoio filantrópico, transferência de tecnologia, plataformas de simulação e integração curricular por sociedades profissionais.

**Conclusões:**

Alcançar a equidade global na cirurgia robótica requer ações coordenadas entre pesquisa, educação, prática clínica, políticas e infraestrutura. A cooperação global e a inovação em estratégias de implementação podem ajudar a superar as disparidades atuais e promover cuidados cirúrgicos seguros e economicamente viáveis em regiões carentes, melhorando os resultados dos pacientes.

## INTRODUCTION

 Robotic surgery represents one of the most significant technological advancements in the field of surgery in the 21st century^17^. Its emergence forms part of a broader continuum of innovation that began with the introduction of laparoscopy in the late 20th century^2^. Just as laparoscopy revolutionized surgical practice for certain operations by enabling minimally invasive procedures with faster recovery times and fewer complications, robotic-assisted surgery has further advanced this evolution, enhancing precision, dexterity, and visualization through computer-assisted platforms. Since the first robotic systems were approved in the early 2000s, robotic surgery has rapidly expanded in certain regions, particularly in high-income countries^19^. 

 However, robotic platforms are considerably more expensive than traditional laparoscopic setups in terms of initial acquisition and maintenance, training, and consumables^22^. Consequently, the global distribution of robotic systems is uneven. Most robotic surgical platforms are situated in North America, Western Europe, and Eastern Asia, with the United States of America (USA) alone hosting more than half of all systems globally. In contrast, many low- and middle-income countries (LMICs) have limited or no access to this technology, exacerbating the surgical care gap between resource-rich and resource-poor regions. 

 For several procedures, including cholecystectomy, appendectomy, and various gynecological and colorectal operations, robotic assistance has yet to demonstrate clear clinical advantages over well-established laparoscopic techniques^9,26,29^. The lack of comprehensive clinical and economic data in LMICs may discourage healthcare administrators from investing in robotic systems where resources are limited, thereby perpetuating global health inequities. 

 While evidence-based medicine remains a cornerstone of clinical decision-making, it is essential to acknowledge its inherent limitations, particularly in rapidly evolving fields such as robotic surgery. High-quality evidence, especially from randomized controlled trials (RCTs), often requires years to generate and is subject to numerous barriers, including funding constraints, regulatory hurdles, and ethical considerations. These challenges can delay the adoption of innovations that may already demonstrate clear advantages in practice^10^. Robotic surgical systems offer advanced features that are challenging to capture fully through traditional study designs. These include enhanced three-dimensional visualization, increased precision, reduced tremors, and ergonomic benefits for surgeons, all of which contribute meaningfully to surgical performance but do not necessarily translate into clinical outcomes or are not easily quantifiable in conventional trials^24^. Moreover, the pace of technological advancement in healthcare now exceeds the speed at which most clinical studies are conducted. This creates a disconnect between the evidence available and the realities of modern practice. For example, by the time a clinical trial on robotic surgery concludes, a new version of the robotic platform may already have been released, rendering the study results less applicable to current technology. In this context, decision-makers must balance the ideal of rigorous evidence with practical considerations about innovation, safety, and clinical benefit. 

 Given this context, it is essential to explore how robotic surgery, as a transformative technology, can be equitably disseminated and integrated worldwide. This integrative review aims to examine the current landscape of robotic surgery diffusion in research, education, and care. It also analyzes and synthesizes global strategies to promote its diffusion, focusing on overcoming barriers in resource-limited settings and providing practical insights that can guide equitable and sustainable implementation worldwide. 

## METHODS

### Study design

 This study is a multinational, policy-oriented integrative review in collaboration with the Research Committee of the Society for Surgery of the Alimentary Tract of the USA (SSAT). The primary objective was to identify, analyze, and synthesize global strategies to enhance the dissemination of research, education, and clinical care in robotic surgery. The methodology integrates expert opinion, bibliometric analysis, and a comprehensive review of the literature to assess the current landscape. 

### Global expert contributions

 Contributions were gathered from SSAT members under the guidance of the SSAT Research Committee, spanning multiple countries, providing regional insights based on their clinical experience, institutional practices, and national health policy contexts. The committee consulted, via email and video conferences, experts in robotic surgery and healthcare leadership to broaden perspectives and validate regional interpretations. This international collaboration ensured a thorough and contextualized assessment of robotic surgery adoption worldwide. 

### Role of the SSAT and its research committee

 The SSAT is a professional society dedicated to advancing the field of gastrointestinal surgery for nearly 70 years. The SSAT promotes scientific excellence, surgical education, and leadership development, aligning with its mission. Its Research Committee is central to identifying emerging challenges and innovations, focusing on producing academically rigorous and policy-relevant work. With a diverse international membership, the committee brings a global perspective to surgical issues, including the equitable dissemination of new technologies such as robotic surgery. 

### Bibliometric analysis and visualization

 To evaluate research trends and diffusion patterns in robotic surgery clinical trials, a bibliometric analysis was performed using data from PubMed. A comprehensive search strategy was employed to maximize sensitivity and specificity, incorporating MeSH terms, free-text keywords, and the names of key robotic platforms. The following search string was used: (("robotic surgical procedures"[MeSH Terms] OR "robotic surgery"[tiab] OR "robot-assisted surgery"[tiab] OR "robotic-assisted surgery"[tiab] OR "robot-assisted"[tiab] OR "robotic-assisted"[tiab] OR "robot surgery"[tiab] OR "surgical robot"[tiab] OR "surgical robotics"[tiab] OR "robotic system"[tiab] OR "robotic platform"[tiab] OR "robotic technique"[tiab]) OR ("Da Vinci"[tiab] OR "Da Vinci Xi"[tiab] OR "Da Vinci X"[tiab] OR "Da Vinci SP"[tiab] OR "DaVinci"[tiab] OR "Intuitive Surgical"[tiab]) OR ("Hugo RAS"[tiab] OR "Hugo™ RAS"[tiab] OR "Medtronic Hugo"[tiab]) OR ("Versius"[tiab] OR "CMR Surgical"[tiab]) OR ("Senhance"[tiab] OR "Senhance Surgical System"[tiab])) AND (clinicaltrial[Filter]). The search was conducted in April 2025 and was limited to clinical trials, with no restrictions on language or publication date. Results were downloaded in PubMed format. 

 Bibliographic data were imported into R (version 4.4.3) and processed using the bibliometrix package, an opensource bibliometric and scientometric analysis tool. The convert2df function was used to parse and structure the PubMed data, followed by biblioAnalysis for the calculation of performance metrics, including annual scientific production and collaborative networks. The biblioshiny interface was used for a visual representation. 

### Additional data sources

 To supplement the bibliometric analysis, a review of gray literature was undertaken. This included governmental health reports, documents from non-governmental organizations, and international health technology assessment publications. Furthermore, qualitative data were derived from structured group discussions within the SSAT Research Committee and consultations with hospital administrators, national surgical leaders, and academic researchers. These sources enriched the contextual interpretation of the bibliometric findings and contributed to a critical appraisal of the diffusion of robotic surgery across different regions. 

## RESULTS

### Robotic care diffusion across the world

**Figure 1:**
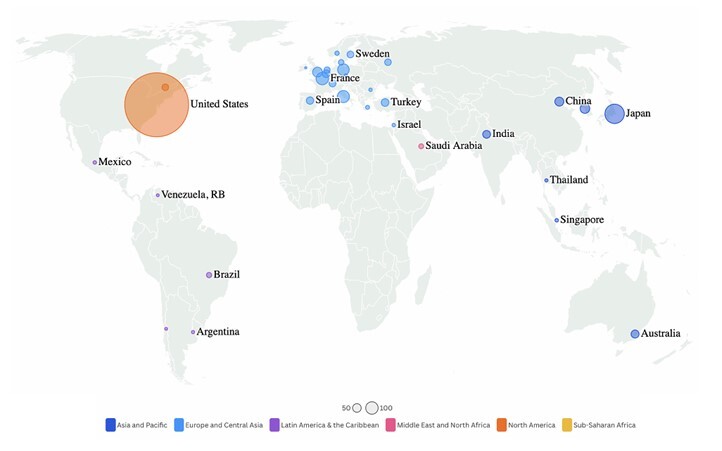
Number of Da Vinci system installations in the world (2015).

 In 1999, the USA had just two Da Vinci systems, while Europe had six. By 2001, the number had increased significantly, with 42 systems installed in the USA and 37 in Europe^30^. According to a 2015 annual report from Intuitive Surgical (Inc.), the Da Vinci Surgical System had been installed in over 6,500 units across 67 countries, with more than 55,000 surgeons trained worldwide. The USA accounted for the vast majority of installations, with 2,344 systems representing 67.4% of all units globally at that time^14^ (Figure 1). 

 Almost 10 years later, in the annual report from Intuitive Surgical on December 31, 2024, the global installed base of Da Vinci surgical systems had reached 9,902 units^13^. Despite this continued expansion, significant geographic disparities in access remain. The USA alone accounts for 5,807 systems, representing nearly 59% of all installations worldwide. In contrast, Europe hosts 1,867 systems, Asia has 1,745, and the rest of the world collectively accounts for only 483 units. This stark imbalance highlights the continued concentration of robotic surgical technology in highincome countries, particularly in the USA, where the penetration of robotic platforms is significantly higher. Even within the USA, there is a significant discrepancy in the distribution of Da Vinci installations, with a predominance on the East Coast (New York and Florida) and the West Coast (California)^30^. 

### Robotic research diffusion around the world

**Figure 2:**
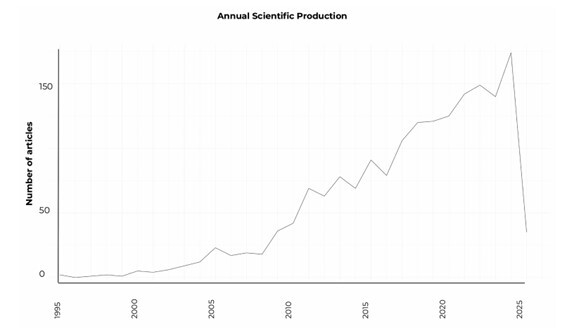
Annual scientific production of clinical trials on robotic surgery.

 A total of 1,758 scientific publications on clinical trials related to robotic surgery were retrieved from 437 sources from 1995 to 2025. The field has shown a notable and consistent expansion, with an average annual growth rate of 10.01% (Figure 2). The analysis identified 9,406 contributing authors, reflecting a high degree of collaboration, as evidenced by an average of 7.8 co-authors per document and an international co-authorship rate of 10.24%. 

**Figure 3:**
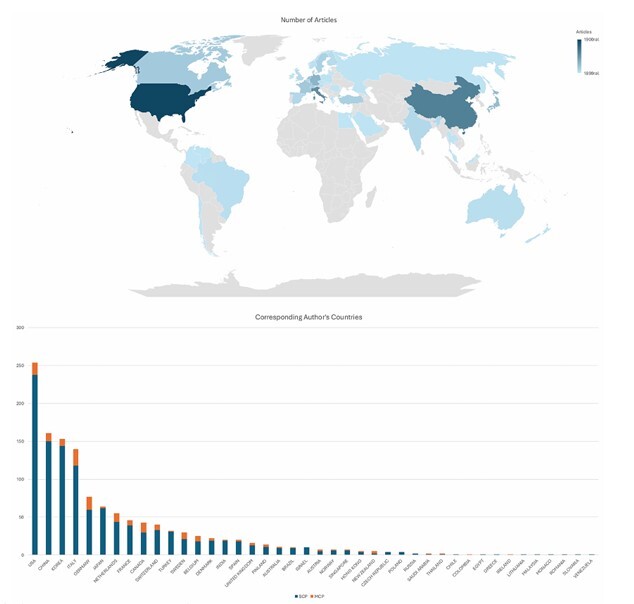
Authors’ countries in trials of robotic surgery. MPC: Multiple-country collaboration; SCP: Single-country collaboration.

**Figure 4:**
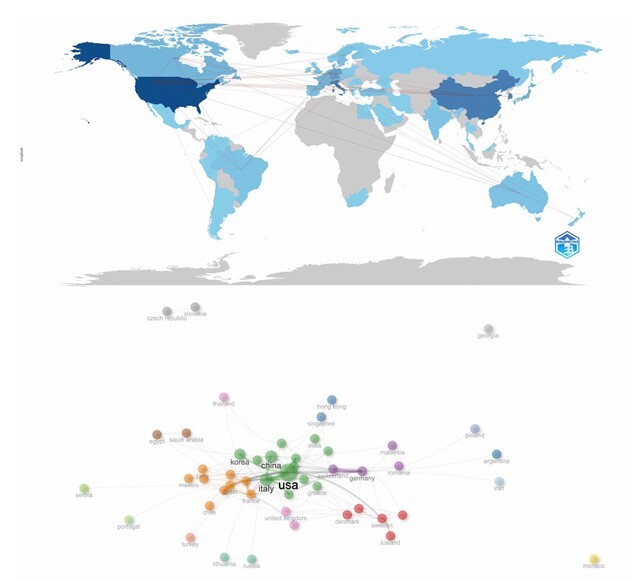
Country collaboration network on trials of robotic surgery. USA: United States of America.

 Despite the growing interest in robotic surgery, the distribution of research output across countries and institutions is unequal. The USA led the field with 254 publications, representing 14.4% of the total, followed by China (161–9.2%), South Korea (153–8.7%), and Italy (140–8.0%) (Figure 3). The country collaboration network further emphasized regional patterns in research influence and integration. For instance, the USA and Italy exhibited the highest betweenness centrality, indicating their pivotal roles in connecting international research clusters (Figure 4). 

**Figure 5:**
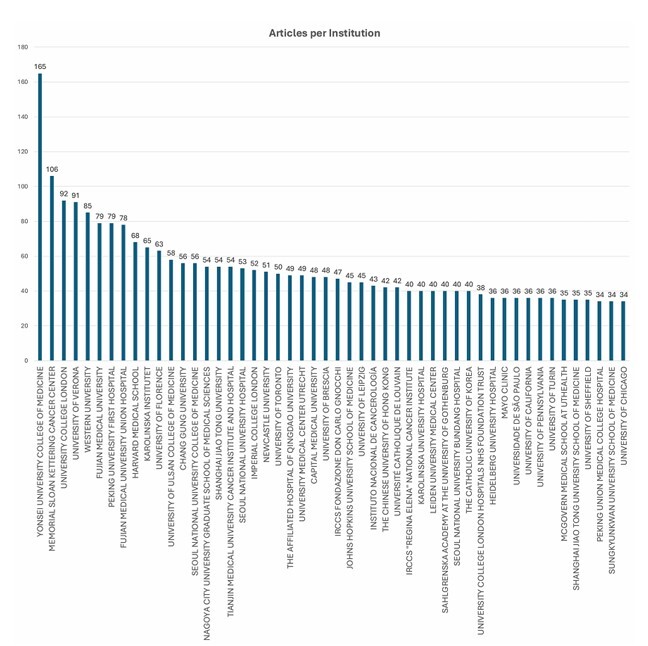
Top 50 most relevant affiliations publishing trials on robotic surgery.

 A handful of academic centers accounted for a disproportionately high number of studies. Yonsei University College of Medicine stood out with 165 publications, far ahead of other institutions such as Memorial Sloan Kettering Cancer Center (106) and University College London (92) (Figure 5). 

 Overall, while robotic surgery is a growing and collaborative field, the production of scientific knowledge remains concentrated in a limited number of countries and institutions, revealing imbalances in research capacity and access across regions. 

### Identifying barriers and facilitators for robotic dissemination

**Figure 6:**
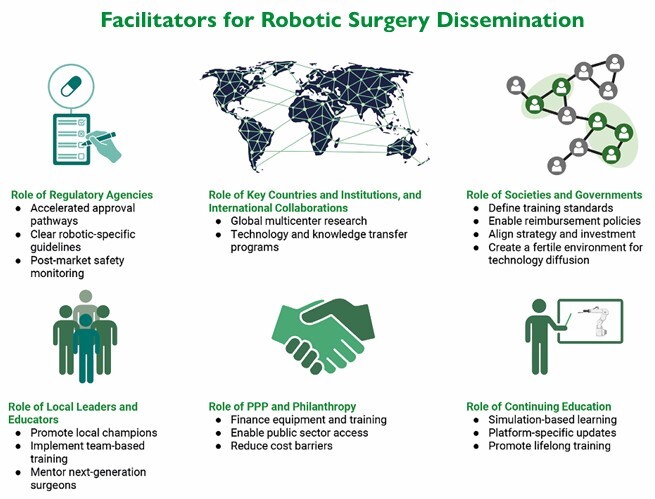
Main facilitators for robotic surgery diffusion. PPP: Public–private partnerships.

 It is essential to examine the challenges and opportunities within multiple levels of the healthcare and innovation ecosystem to better understand the complexities involved in the global dissemination of robotic surgery (Figure 6). These levels encompass the domains of the researcher, educator, surgeon, institution, and society. Each of these areas presents distinct barriers that can impede progress, as well as specific facilitators that can promote broader and more equitable adoption of robotic surgical technologies. By systematically identifying and addressing these factors, stakeholders can formulate more targeted strategies for implementation and scale-up. A detailed summary of these barriers and facilitators appears in Table 1. 

**Table 1:** Summary of key barriers and facilitators for disseminating robotic surgery, organized by level of influence: Researcher, educator, surgeon, and institutional/societal.

	Researcher level	Educator level	Surgeon level	Institutional/societal level
Barrier	• High costs of conducting RCTs for robotic surgery, particularly in LMICs	• Absence of robotic surgery experts to serve as trainers in new programs	• Psychological barriers: Resistance to change, fear, skepticism, technophobia	• Infrastructure limitations (e.g., small ORs, narrow doors, and insufficient power supply)
	• Ethical concerns regarding trials involving unapproved robotic systems	• Lack of institutional support for developing robotic curricula	• Perceived loss of human interaction and tactile feedback	• High cost of acquisition and maintenance of robotic platforms
	• Regulatory barriers that delay trial initiation and data publication	• Limited availability of simulation labs and robotic systems for teaching	• Difficulties in understanding complex robotic systems	• Lack of reimbursement frameworks and unclear costeffectiveness analysis
	• Lack of standardized research frameworks to evaluate effectiveness and safety	• Variability in educational standards and learning objectives	• Older surgeons may feel less motivated or confident in adopting technology	• Complex regulatory environments delaying approval and access
	• Limited incentives for independent comparative effectiveness research	• Lack of unified certification or accreditation pathways for robotic surgery education	• Low literacy or unfamiliarity with new systems	• Workforce challenges in coordinating multidisciplinary robotic teams
	• Disparities in access to research funding		• Disruption in workflow and learning curve stress	• Unequal access to advanced versions of robotic systems
			• Lack of motivation due to perceived redundancy or irrelevance	• Fragmented training efforts without cohesive national planning
Facilitator	• Partnerships between academia and industry to support trials	• Creation of formal curricula	• Continuing education and exposure to evidence on safety and outcomes	• PPP to finance platforms and training
	• Use of observational studies, real-world data, and registries to complement RCTs	• Use of simulation platforms, remote learning, and virtual proctoring	• Involvement in co-development and clinical validation of new systems	• Involvement of surgical societies in defining standards and certifying competence
	• Support from national and international research agencies	• International fellowships and training	• Intuitive interface design and ergonomic advantages	• Government grants, tax incentives, and regulatory streamlining
	• Development of open-access data platforms and global research consortia	• Mentorship networks to build teaching capacity	• User training programs and hands-on workshops	• Regulatory agency reforms to accelerate safe adoption
	• Integration of robotic research into national innovation policies	• Curriculum modularization to train the entire surgical team	• Peer influence and robotic champions in surgical departments	• Capacity-building: Fellowships, simulation centers, and team-based curricula
	• Government investment in robotic research programs	• Recording and auditing tools for performance assessment	• Demonstrated benefits like reduced morbidity and shorter hospital stays	• Philanthropic support to overcome cost barriers and promote access
				• Strategic policy planning with modular tech transfer models

RCTs: Randomized controlled trials; LMICs: Low- and middle-income countries; OR: Operating room; PPP: Public–private partnerships.

### Researcher-level barriers and facilitators for robotic surgery dissemination

 One of the most significant challenges is the high cost of conducting clinical trials, particularly RCTs, which demand substantial infrastructure, specialized personnel, and long-term follow-up. In addition, ethical concerns often arise in RCTs, especially in studies involving new or upgraded robotic systems that have not yet received approval from local regulatory agencies. These concerns, combined with bureaucratic complexity and lengthy approval processes, can significantly delay the initiation of studies and hinder the timely generation of evidence needed to support the adoption of innovative technologies. 

 Despite these challenges, a range of facilitators has emerged to support and accelerate the development of robotic surgery research. One promising strategy is promoting translational research through animal and cadaveric models, which enables the early testing of new or upgraded robotic systems in controlled environments, thereby minimizing ethical concerns related to human risk while generating valuable preliminary data. Additionally, integrating observational studies and big data analytics can supplement traditional clinical trials, helping to fill critical evidence gaps. These types of approaches enable access to larger and more diverse patient populations, providing more generalizable data that may ultimately facilitate the globalization of robotic surgery. Clinicians performing high-volume robotic surgery should be encouraged to maintain well-organized clinical registries, allowing for ongoing outcomes, research, and health technology assessments. These data, if collected throughout the lifecycle of a technology, can provide valuable insights into clinical efficacy and cost-effectiveness. Numerous international registries, such as the Upper Gastrointestinal International Robotic Association (UGIRA), the International Robotic and Laparoscopic Liver Resection Study Group, and the European Consortium on Minimally Invasive Pancreatic Surgery, compile robotic data from worldwide surgeons, generating large datasets that become a substantial source for research and statistical analysis^16,18,20^. 

 A key enabler of research dissemination is the development of multinational and multi-institutional studies, which facilitate the pooling of expertise, infrastructure, and funding from leading centers worldwide. Highly productive countries and institutions, particularly those in the USA, Italy, and China, play a significant role in advancing and disseminating robotic surgery research. Their leadership helps establish scientific standards, foster global collaborations, and accelerate the adoption of robotic technologies across diverse health-care settings. 

 Governments play a central and strategic role in shaping healthcare systems. They are ultimately responsible for managing public health resources and deciding which technologies are worth adopting in public care. The government’s role in technology transfer is to reduce barriers by implementing policies such as intellectual property rights, neutral taxation, and limited regulation^4^. 

 However, funding may be directed toward specific objectives when research aligns closely with political or national priorities. Public support for high-quality research remains essential. Robust scientific evidence is crucial for determining which surgical methods, including robotic-assisted techniques, yield the best outcomes in terms of cost-effectiveness, safety, and patient care. In Brazil, a series of randomized trials was conducted by the São Paulo Cancer Institute (Instituto do Câncer do Estado de São Paulo — ICESP), funded by the state government (ReBEC: RBR-5s6mnrf; ClinicalTrials.gov: NCT02292914). These studies evaluated the safety, effectiveness, and costs of robotic surgery within the Brazilian public health system and now represent several of the country’s leading publications on robotic surgery. This case illustrates how targeted public investment in research can produce the evidence needed to support informed, data-driven healthcare decisions. 

### Educator-level barriers and facilitators for robotic surgery dissemination

 At the educator level, the dissemination of robotic surgery faces several key obstacles. A significant barrier is the lack of qualified experts to initiate and lead training programs in many institutions, particularly in regions where robotic surgery is still in its early stages of development. Without experienced faculty, it becomes difficult to establish structured educational pathways or build institutional credibility in robotic training. Variability in training standards, the absence of unified certification pathways, poor coordination between academic centers, and a lack of institutional support also impair the development or integration of robotic surgery into existing curricula. 

 Despite these limitations, several facilitators can drive progress, primarily through capacity-building strategies in education and leadership. Sustainable technology diffusion requires investment in human capital. Training programs, mentorship networks, and the development of regional centers of excellence can foster local expertise and reduce dependency on external actors. Promoting local clinical leaders and robotic champions accelerates robotic dissemination in multiple settings. Pettersen et al.^23^, in a systematic review, highlighted the key role of local champions in promoting the adoption of new technologies in healthcare. They identified two main types: management-level and clinical-level champions. Management-level champions are involved in planning and advising, using their clinical insights to align implementation with everyday clinical workflows. Clinical-level champions, on the other hand, are crucial for the hands-on integration of technology into daily practice. They participated in discussions with senior management and influenced peers. Consequently, the authors conclude that a single champion per institution is insufficient, and a set of leaders at various touchpoints in the complex healthcare ecosystem is needed to promote technology diffusion. 

 Countries with established leadership in robotic surgery, such as the USA and Italy, and major academic institutions, play a crucial role in global dissemination efforts. By sharing expertise with smaller or less-resourced institutions, these centers can serve as regional hubs for education, mentorship, and capacity-building. Successful strategies include developing formal curricula and integrating simulation-based training, remote learning, and virtual proctoring, which help overcome geographical and resource limitations while ensuring high-quality education. Structured fellowship programs, virtual learning platforms, and partnerships with international surgical societies have the potential to expand access to robotic surgery training, particularly in regions with limited simulators and robotic platforms. These fellowship programs should aim not only for technical expertise but also for managerial-level skills, enabling the successful implementation of these technologies once fellows return to their respective institutions. 

 In this context, local and international societies, such as SSAT, play a pivotal role in guiding structured fellowship programs, virtual learning platforms, and partnerships with international surgical societies that have effectively extended robotic training to regions with limited access to simulation centers or robotic platforms. SSAT promotes international webinars, "how-I-do" videos, mentorship initiatives, and visiting professorship awards to foster global knowledge exchange and surgical education. Other successful strategies that local and international societies can promote include the development of formal curricula, structured fellowship programs, remote learning platforms, and virtual proctoring. Formal education alone does not significantly improve international technology transfer. Success is more strongly linked to targeted training in technology transfer-related areas than to the number of highly educated staff^3^. 

 The United Kingdom has adopted a structured and governance-driven approach to integrating robotic-assisted surgery, primarily led by the Royal College of Surgeons of England^27^. Recognizing the transformative potential of robotic technologies, the College has positioned itself at the forefront of ensuring that robotic surgery is implemented safely, ethically, and with high-quality training standards. The College actively promotes the use of less complex procedures for robotic training when appropriate, thereby facilitating skill acquisition in a controlled and educationally beneficial environment. 

### Surgeon-level barriers and facilitators for robotic surgery dissemination

 A combination of personal and psychological barriers can significantly slow the adoption of robotic surgery. Resistance to change, technophobia, and skepticism about the value of robotic systems are particularly common among experienced surgeons, who may be less inclined to adopt unfamiliar technologies. One frequent concern is the absence of tactile feedback, which can reduce a surgeon’s confidence in the system. Additionally, there is concern about managing emergencies, such as sudden bleeding, where prompt intervention is crucial. This concern is heightened during the early stages of a surgeon’s robotic learning curve, when undocking may take longer and complications may be more common^15^. 

 Research on the dissemination of digital health technologies highlights the challenges many professionals face in mastering complex systems, as well as anxiety related to steep learning curves^32^. These issues, combined with low expectations, fear of failure, and the disruption of familiar workflows, can significantly reduce motivation and delay the integration of robotic platforms into routine practice. In addition, skepticism persists regarding the incremental clinical benefits of robotic surgery for many procedures, particularly when compared with established minimally invasive techniques. This skepticism is amplified by the high costs involved, which in most countries extend beyond acquisition and maintenance to substantial case-by-case expenses for disposable instruments and consumables. Together, these technical, perceptual, and financial barriers create significant resistance to adoption, especially in resource-constrained environments. 

 Despite these challenges, several key facilitators can improve surgeon engagement and encourage broader adoption. Ongoing education, practical training, simulation-based learning, and exposure to high-quality evidence can help alter attitudes and boost confidence. The presence of robotic champions within departments, supported by peer mentorship and collaborative learning environments, can serve as a motivating factor. 

 In critical emergency undocking situations, outcomes may vary depending on the clinician’s experience and training. Establishing and routinely practicing standardized emergency protocols can reduce this variability, ensuring prompt and coordinated responses, improving patient safety, and increasing confidence in robotic operations^28^. 

 Advancements in new or upgraded robotic platforms, such as improved docking mechanisms, streamlined workflows, emerging haptic feedback technologies, and user-friendly ergonomic systems, offer additional reassurance for surgeons, making these systems more straightforward and safer. 

### Institutional-level barriers and facilitators for robotic surgery dissemination

 Various structural, financial, and strategic factors influence the dissemination of robotic surgery. Several barriers persist, particularly in lower-resourced settings. Infrastructure is one of the most immediate limitations. Many hospitals lack operating rooms with the appropriate size, layout, or electrical systems to accommodate robotic platforms. Narrow doorways, insufficient space for docking, and the absence of dedicated simulation centers or training environments further complicate the implementation of robotics. Additionally, several studies have shown that robotic surgery is associated with longer operative times, mainly due to docking and setup^31^. This makes surgery scheduling challenging and more costly in already-busy operating rooms. 

 Another major obstacle is the high costs associated with the robotic procedures. Robotic platforms represent a substantial investment, not only in their initial acquisition but also in ongoing maintenance, consumables, and staffing. Studies from multiple countries consistently show that robotic surgery is more expensive than traditional laparoscopic procedures. In China, a study comparing robotic and laparoscopic colorectal surgeries found that robotic cases were, on average, USD 2,258.80 more expensive^12^. A USA national analysis also found that robotic procedures were consistently more costly than laparoscopic ones, with the gap increasing over time, from USD 1,600 in 2012 to USD 2,600 in 2019^21^. Similarly, in Brazil, robotic incisional hernia repairs cost R$ 14,712.24, compared to R$ 10,295.95 for laparoscopic repairs, due to significantly higher operating room time, human resources, and consumables expenses^8^. 

 Compounding this issue is the rapid evolution of robotic technology. Introducing new and upgraded systems, such as the Da Vinci 5, adds further financial strain. These newer models feature improved workflow integration, enhanced docking capabilities, and haptic (tactile) feedback, addressing a long-standing limitation of earlier systems. However, their high cost has limited access to a few high-income institutions; only 362 units had been installed worldwide as of late 2024, highlighting the growing divide not just in access to robotic surgery but also in its most advanced systems^13^. 

 Another structural challenge is the lack of reimbursement mechanisms for robotic procedures in many countries. In Brazil, for instance, several private insurance companies and the public health system (Unified Health System [SUS]) do not reimburse robotic surgeries, making it difficult for public and small private institutions to justify the investment. In the USA, hospitals often operate with tight financial margins, and reimbursement rates from Medicare and Medicaid are typically limited^33^. This creates a challenging environment for integrating robotic surgery, as robotic instruments and maintenance costs must be absorbed within fixed payment models. Unless minimal-cost systems are used, hospitals may face financial losses per case. Therefore, to be economically feasible, robotic platforms must be paired with clear financial benefits or revenue-generating advantages. 

 Despite these challenges, several facilitators have emerged to support institutional adoption and overcome resource constraints. Public–private partnerships (PPPs) are promising, as they enable shared investment in training, technology, and service provision. A notable example is the Hospital Municipal Vila Santa Catarina, Hospital Israelita Albert Einstein model, in São Paulo, Brazil, where surgeons from private institutions fund their robotic training in public hospitals, while public patients benefit from the procedures under ethical oversight. This model expands access to training and creates a pathway for early adoption of robotics in the public sector. 

 Philanthropy has also played a key role in overcoming financial barriers. The case of the Hospital de Amor (formerly Hospital de Câncer de Barretos) demonstrates how tax-incentivized donations—through initiatives such as Brazil’s PRONON program—can make high-cost technologies accessible to underserved populations^6^. Despite acquiring a Da Vinci robot system in 2013, the hospital faced a 1-year delay in implementing it due to the high maintenance costs, estimated at R$3.5 million over 10 years, which was also eventually covered by the same philanthropic initiative. Continued financial backing allowed the program to succeed; surgeries that would otherwise be unaffordable for public patients became possible. 

### Societal-level barriers and facilitators for robotic surgery dissemination

 The interplay of governments, surgical societies, regulatory agencies, and broader healthcare policies shapes the dissemination of robotic surgery. A primary concern among policymakers and healthcare administrators is the risk of inefficient resource allocation. Robotic platforms are significantly more expensive than conventional alternatives such as laparoscopy, yet their clinical advantages may not always be clearly demonstrated in cost-effectiveness analyses^11,25,31^. This raises skepticism about whether public funds should be invested in such high-cost technologies, particularly in resource-constrained healthcare systems. 

 Governments play a pivotal role in creating an environment that enables the responsible and equitable evolution of robotic surgery. This involves direct investments in infrastructure, education, and research, as well as indirect support through tax incentives, streamlined regulatory processes, and innovation-focused policy frameworks. Government funding for cost-effectiveness trials, particularly those conducted in public systems, is crucial for guiding rational adoption. 

 Beyond funding, governments must also invest in regulatory modernization. Agencies such as the Food and Drug Administration (FDA), the European Medicines Agency (EMA), and the Agência Nacional de Vigilância Sanitária (ANVISA) play a crucial role in determining how quickly new robotic systems reach clinical settings. Prolonged and inconsistent regulatory processes, although necessary to ensure safety, can delay access and widen global disparities. Countries that strike a balance between rigorous evaluation and agility are better positioned to integrate robotic platforms efficiently. 

 The FDA plays a leading role in the USA. Intuitive Surgical introduced the first generation of the Da Vinci Surgical System in 1999, and it received FDA approval in 2000 for general surgery, making it the first robotic platform authorized^7^. However, approval for additional procedures, such as urological and oncologic surgeries, was granted incrementally in subsequent years as further clinical evidence was submitted. The FDA’s early and structured endorsement was pivotal to the Da Vinci system’s rapid adoption and eventual dominance in the robotic surgery market. 

 The regulatory approval process for robotic systems is typically complex and time-consuming, as agencies must thoroughly evaluate safety, effectiveness, and clinical benefit. However, this process also hampers the introduction of new clinical trials supporting their use in practice. This extended timeline can significantly delay access to new robotic technologies and upgraded versions of existing systems in certain regions. 

 In Europe, the EMA and the European Commission oversee the approval of medical devices through the *Conformité Européenne* (CE) marking process. This system is often fast but places more responsibility on manufacturers for post-market surveillance. For instance, the Hugo RAS system by Medtronic received CE Mark approval in 2022 for gynecologic and urologic procedures, allowing it to enter the European market before gaining clearance in the USA. The Versius Robotic System, developed by CMR Surgical (Cambridge Medical Robotics), gained CE Mark approval in Europe before expanding into other regions. It only received FDA clearance in 2024^5^. 

 In Brazil, ANVISA regulates medical technologies. In 2024, ANVISA signed a mutual confidentiality agreement with the FDA, strengthening collaboration between the two agencies. This agreement enables the exchange of confidential, non-public information regarding regulated products. This type of alignment has the potential to make regulatory systems more dynamic and responsive while maintaining high safety and efficacy standards^1^. 

 The successful dissemination of robotic surgery depends not only on governmental action but also on the engagement of surgical societies and academic institutions, which play a central role in shaping education, policy, and clinical standards. Organizations like the SSAT and national surgical associations serve as key intermediaries between clinicians, healthcare institutions, and policymakers. Their contributions include advocating for the inclusion of robotic surgery in national training curricula, developing competency frameworks, and establishing credentialing standards for individual surgeons and institutions. These initiatives are crucial to ensuring patient safety, professional accountability, and broader acceptance of robotic technologies. 

 Moreover, these societies facilitate international knowledge exchange through technology transfer strategies, such as licensing agreements, joint ventures, and regional research collaborations. These mechanisms allow the sharing of innovation and expertise across borders, accelerating the development and local adaptation of robotic surgery programs, particularly in emerging healthcare markets. 

 Ultimately, aligning government policies, surgical societies, and academic bodies is essential to creating a supportive ecosystem for robotic surgery. When these stakeholders collaborate effectively, they can foster cost-efficient implementation, enhance surgical training and team readiness, and ensure that robotic platforms deliver meaningful value to both healthcare providers and patients. 

## DISCUSSION

 This review highlights the multifaceted strategies that support the global diffusion of robotic surgery. Key enablers include PPPs, government engagement in funding and policymaking, investment in education, and local leadership, as well as systemic efforts to promote innovation and technology transfer. The role of governments and surgical societies in creating a supportive legal, institutional, and financial environment is especially crucial, as is the development of collaborative models that integrate local expertise with global technology platforms. The review also emphasizes the importance of modularity and infrastructure as emerging solutions to reduce entry barriers and promote scalability in resource-limited settings. 

 The current literature on robotic surgery has several limitations. One major challenge is the limited availability of clinical studies assessing the implementation and outcomes of robotic surgery in low-resource environments. Most published data originate from high-income countries, making it challenging to generalize findings or assess the actual feasibility of under-resourced health systems. Additionally, there is a lack of robust cost-effectiveness analyses comparing robotic surgery to traditional and laparoscopic techniques in low-income settings. These gaps hinder evidence-based policy decisions and slow the formulation of national strategies for integrating robotic surgery into public healthcare systems. 

 Future research should prioritize prospective studies in LMICs, focusing on clinical outcomes, financial sustainability, and system-level impact. Cost-effectiveness analyses are particularly needed to guide policymakers and payers in determining when and how robotic surgery offers added value. Furthermore, there is a pressing need to concentrate on the most prevalent surgical conditions where robotic assistance might offer measurable improvements in safety, recovery, or long-term outcomes. 

 Looking ahead, the next generation of robotic platforms holds promise for expanding access. These new robotic systems may increase market competition, reduce dependency on proprietary systems, and stimulate regional innovation ecosystems. Strategic investment in training, coupled with evaluation frameworks tailored to local contexts, will be critical to ensure that the expansion of robotic surgery aligns with the principles of equity, efficiency, and global surgical advancement. 

## CONCLUSIONS

 Achieving global equity in robotic surgery requires coordinated action across research, education, clinical practice, policy, and infrastructure. International cooperation and innovation in implementation strategies can help bridge the current disparities and promote safe, cost-effective surgical care in underserved regions. 

## Data Availability

The Informations regarding the investigation, methodology and data analysis of the article are archived under the responsibility of the authors.

## References

[1] Agência Nacional de Vigilância Sanitária (ANVISA) (2024). Anvisa e FDA assinam acordo de confidencialidade [Internet].

[2] Alkatout I, Mechler U, Mettler L, Pape J, Maass N, Biebl M (2021). The development of laparoscopy-a historical overview. Front Surg.

[3] Araújo C, Teixeira A (2014). Determinants of international technology transfer: An empirical analysis of the Enterprise Europe Network. J Technol Manag Innov.

[4] Bozeman B (2000). Technology transfer and public policy: a review of research and theory. Res Policy.

[5] Brassetti A, Ragusa A, Tedesco F, Prata F, Cacciatore L, Iannuzzi A (2023). Robotic surgery in urology: history from PROBOT® to HUGOTM. Sensors (Basel).

[6] Brazil. Ministry of Health PRONON and PRONAS/PCD: Support Programs for Oncology Care and People with Disabilities.

[7] Chopra H, Baig AA, Cavalu S, Singh I, Emran TB (2022). Robotics in surgery: Current trends. Ann Med Surg (Lond).

[8] Costa TN, Tustumi F, Ferros LSM, Colonno BB, Abdalla RZ, Ribeiro-Junior U (2023). Robotic-assisted versus laparoscopic incisional hernia repairs: differences in direct costs from a Brazilian public institute perspective. Arq Bras Cir Dig.

[9] Delgado LM, Pompeu BF, Pasqualotto E, Magalhães CM, Oliveira AFM, Kato BK (2024). Robotic-assisted cholecystectomy versus conventional laparoscopic cholecystectomy for benign gallbladder disease: a systematic review and meta-analysis. J Robot Surg.

[10] Einav S, O’Connor M (2024). The limitations of evidence-based medicine compel the practice of personalized medicine. Intensive Care Med.

[11] Gkegkes ID, Mamais IA, Iavazzo C (2017). Robotics in general surgery: A systematic cost assessment. J Minim Access Surg.

[12] Hu DP, Zhu XL, Wang H, Liu WH, Lv YC, Shi XL (2021). Robotic-assisted *versus* conventional laparoscopic surgery for colorectal cancer: Short-term outcomes at a single center. Indian J Cancer.

[13] Intuitive Surgical, Inc (2024). 2024 Annual Report.

[14] Intuitive Surgical (2015). da Vinci’s manufacturer. Investor Presentation.

[15] Kassite I, Bejan-Angoulvant T, Lardy H, Binet A (2019). A systematic review of the learning curve in robotic surgery: range and heterogeneity. Surg Endosc.

[16] Kooij CD, de Jongh C, Kingma BF, van Berge Henegouwen MI, Gisbertz SS, Chao YK (2025). The current state of robot-assisted minimally invasive esophagectomy (RAMIE): outcomes from the upper GI International Robotic Association (UGIRA) esophageal registry. Ann Surg Oncol.

[17] Lane T (2018). A short history of robotic surgery. Ann R Coll Surg Engl.

[18] Li Z, Pfister M, Raptis DA, Ma Y, Yang G, Li L (2025). Novel benchmark for robotic liver resection - bridging tradition with innovation. Ann Surg.

[19] Morrell ALG, Morrell-Junior AC, Morrell AG, Mendes JMF, Tustumi F, De-Oliveira-e-Silva LG (2021). The history of robotic surgery and its evolution: when illusion becomes reality. Rev Col Bras Cir.

[20] Müller PC, Breuer E, Nickel F, Zani S, Kauffmann E, De Franco L (2023). Robotic distal pancreatectomy: a novel standard of care? Benchmark values for surgical outcomes from 16 international expert centers. Ann Surg.

[21] Ng AP, Sanaiha Y, Bakhtiyar SS, Ebrahimian S, Branche C, Benharash P (2023). National analysis of cost disparities in robotic-assisted versus laparoscopic abdominal operations. Surgery.

[22] Peng Y, Liu Y, Lai S, Li Y, Lin Z, Hao L (2023). Global trends and prospects in health economics of robotic surgery: a bibliometric analysis. Int J Surg.

[23] Pettersen S, Eide H, Berg A (2024). The role of champions in the implementation of technology in healthcare services: a systematic mixed studies review. BMC Health Serv Res.

[24] Probst P (2023). A review of the role of robotics in surgery: to DaVinci and beyond!. Mo Med.

[25] Ramsay C, Pickard R, Robertson C, Close A, Vale L, Armstrong N (2012). Systematic review and economic modelling of the relative clinical benefit and cost-effectiveness of laparoscopic surgery and robotic surgery for removal of the prostate in men with localised prostate cancer. Health Technol Assess.

[26] Reddy S, Tote D, Zade A, Sudabattula K, Dahmiwal T, Hatewar A (2024). Comparative analysis of robotic-assisted versus laparoscopic appendectomy: a review. Cureus.

[27] Royal College of Surgeons of England Robotic-assisted surgery: a good practice guide.

[28] Shah SB, Chawla R, Rawal SK (2023). Rapid undocking protocol for the Da Vinci surgical robot during emergency situations. Indian J Anaesth.

[29] Solaini L, Bocchino A, Avanzolini A, Annunziata D, Cavaliere D, Ercolani G (2022). Robotic versus laparoscopic left colectomy: a systematic review and meta-analysis. Int J Colorectal Dis.

[30] Vaessen C (2011). Location of robotic surgical systems worldwide and in France. J Visc Surg.

[31] Walshaw J, Huo B, McClean A, Gajos S, Kwan JY, Tomlinson J (2023). Innovation in gastrointestinal surgery: the evolution of minimally invasive surgery-a narrative review. Front Surg.

[32] Whitelaw S, Pellegrini DM, Mamas MA, Cowie M, Van Spall GC (2021). Barriers and facilitators of the uptake of digital health technology in cardiovascular care: a systematic scoping review. Eur Heart J Digit Health.

[33] Yim NH, McCarter J, Haykal T, Aral AM, Yu JZ, Reece E (2023). Robotic surgery and hospital reimbursement. Semin Plast Surg.

